# Novel modality using computational fluid dynamics to estimate renal pelvic pressure and evaluate severity of obstruction in congenital hydronephrosis

**DOI:** 10.3389/fruro.2025.1634278

**Published:** 2025-09-09

**Authors:** Kenichi Nishimura, Syuta Imada, Naoya Sugihara, Tetsuya Fukumoto, Noriyoshi Miura, Yuki Miyauchi, Tadahiko Kikugawa, Masanori Nakamura, Takashi Saika

**Affiliations:** ^1^ Department of Urology, Ehime University Graduate School of Medicine, Toon, Ehime, Japan; ^2^ Department of Mechanical Engineering, Nagoya Institute of Technology, Nagoya, Aichi, Japan

**Keywords:** hydronephrosis, renal pelvic pressure, pyeloplasty, urine output, surgical indications

## Abstract

**Background:**

Congenital hydronephrosis involves obstruction of the ureteropelvic junction, which impairs urine passage and elevates renal pelvic pressure. Elevated renal pelvic pressure detrimentally affects renal function. Pyeloplasty is a surgical procedure that aims to prevent deterioration of renal function. The Whitaker test, which is conducted using nephrostomy, is used to measure renal pelvic pressure. However, this method is highly invasive, highlighting the need for alternative testing approaches. Computational fluid dynamics (CFD) provides quantitative predictions of fluid flow phenomena and has recently been applied in medicine.

**Objective:**

The aim of this study was to develop an evaluation method using computational fluid dynamics (CFD) to determine pyeloplasty indications.

**Methods:**

The CFD were analyzed using computed-tomography-extracted images. The urine flow in the extracted geometry was simulated by solving the continuity and Navier–Stokes equations.

**Key findings and limitations:**

CFD analysis revealed that renal pelvic pressure increases when urine output increases because of ureteropelvic junction obstruction during hydronephrosis. Furthermore, hydronephrosis with a renal pelvic pressure of 0.015–0.086 Pa, within the physiological urine output range of 360–1440 mL/day, was associated with poor renal function. The main limitation of this method is that the intrarenal pressure analyzed using CFD is an estimate and not the actual pressure.

**Conclusions and clinical implications:**

Thus, renal-pelvic pressure can be measured through CFD analysis. Furthermore, CFD analysis can be used as a new modality to determine severity of obstruction.

## 1 Introduction

Hydronephrosis is a commonly encountered clinical condition. Congenital hydronephrosis involves obstruction of the ureteropelvic junction, resulting in urinary stasis and elevated pressure on the renal pelvis. High renal pelvic pressure impairs urine production in the renal parenchyma, causing direct renal injury. Moreover, prolonged and severe hydronephrosis results in irreversible renal dysfunction (1–3).

Ultrasonography is a tool used for examining various morphological aspects. The hydronephrosis grade and anteroposterior diameter of the renal pelvis are indicators of pyeloplasty (4, 5). However, whether hydronephrosis grade or anteroposterior diameter is associated with future renal function remains unclear. Moreover, the ability of ultrasonography to estimate and predict changes in renal function is limited. Diuretic renograms can be used to evaluate relative renal function and obstruction as well as current renal function, but cannot be used to predict future renal function. Furthermore, diuretic renograms have several limitations, including complicated procedures and limited availability of facilities. is an antegrade method for measuring renal pelvic pressure and remains the only test that directly quantifies pressure within the renal pelvis.

The Whitaker test, performed using nephrostomy, is an antegrade method for measuring renal pelvic pressure and is the only test that directly quantifies pressure within the renal pelvis (6). This test is crucial to determine the severity of hydronephrosis. However, this test is invasive and non-physiological owing to the diffusion of pressure by nephrostomy and muscle contraction by stimulation, therefore, it is rarely performed. The Whitaker test cannot be used for determining the surgical indications for hydronephrosis. Moreover, no noninvasive tests that estimate renal pelvic pressure are currently available, highlighting the need for a novel examination method to determine the indications for pyeloplasty.

Computational fluid dynamics (CFD) is a method developed using computers to quantitatively predict fluid mechanical phenomena based on conservation laws (conservation of mass, momentum, and energy). CFD is a promising noninvasive technique for observing and collecting flow information (7). CFD can be used for analyzing vesicoureteral reflux during stent insertion as well as preoperative and postoperative posterior urethral valves; however, no reports have been published on using CFD for analyzing intrarenal pelvic pressure (8, 9). We hypothesized that CFD could serve as an alternative to the Whitaker test for quantifying the pressure difference during renal pelvic ureteral junction stenosis and for non-invasively determining the functional severity of stenosis. We aimed to develop a novel method using CFD analysis to evaluate severity of obstruction in congenital hydronephrosis.

## 2 Methods

**Figure 1:**
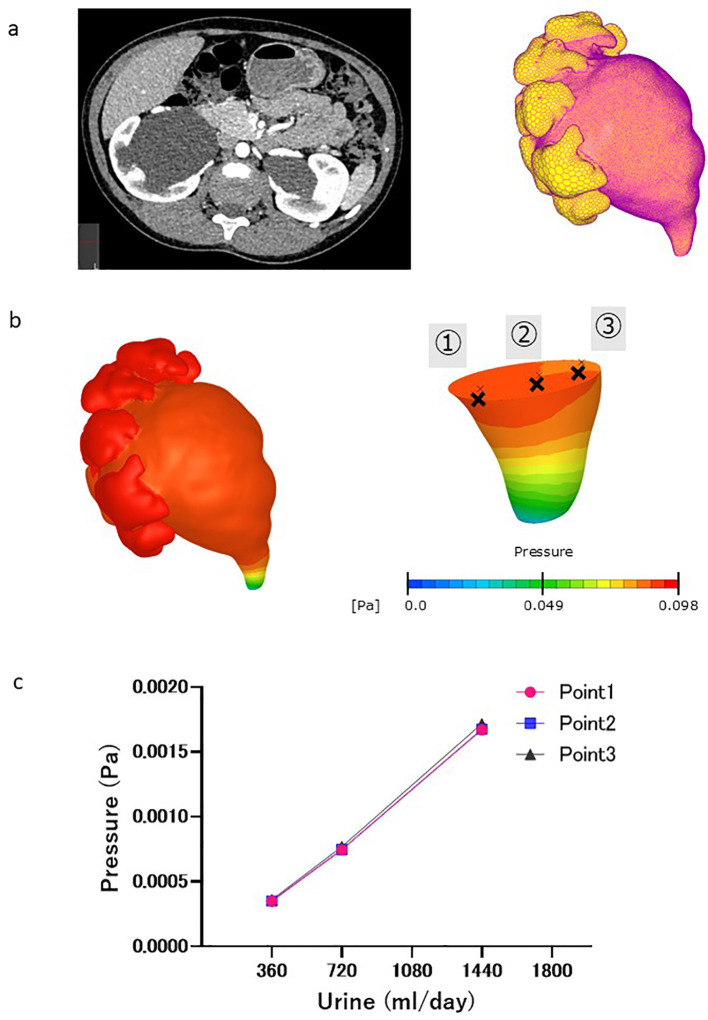
CFD analysis procedure. **(a)** Left: Contrast-enhanced computed tomography (CT) images of the abdomen (cross-sectional view) revealed severe hydronephrosis and congenital ureteropelvic junction stenosis in the right kidney. Right: L Luminal geometry of the renal pelvis and ureter, extracted from CT images, as well as the inflow boundary condition (yellow area). **(b)** Pressure distribution in renal pelvis at ureter outlet pressure of 0 Pa. Pressure at different fault plane sites were calculated (points ①, ②, and ③). **(c)** Change in pressure difference associated with virtual urine flow rate. No pressure differences were observed at different fault plane sites (points ①,②, and ③).


The CFD analysis procedure was as follows. The luminal geometries of the renal pelvis and ureter were extracted from the computed tomography (CT) images (**Figure 1a**, left). The luminal geometries were divided into polyhedral meshes via allocating two layers of prism meshes to the surface (**Figure 1a**, right). A total number of meshes was approximately one million. An inflow boundary condition was applied to the papillary region of the renal pelvis. The urinary flow in the extracted geometry was simulated using scFlow 2023 (MSC software, Japan) by solving the continuity and Navier–Stokes equations:

(1) ∇·U=0

(2) $$\rho \left(U\cdot \nabla \right)U=-\nabla P+\mu \nabla^{2}U$$

where **U** is the three-dimensional velocity vector; *P* is the pressure; and *ρ* and *μ* are the density and dynamic viscosity of blood, respectively. Urine was assumed to be an incompressible Newtonian fluid with a density of 1050 kg/m^3^ and viscosity of 0.001 Pa·s (10). Urinary flow rates of 0.25 to 1.0 mL/min were applied based on the typical daily urinary volume of humans from infancy to adolescence. The pressure at the ureteral outlet was set at 0 Pa. A no-slip condition was applied to the walls of the renal pelvis and ureter assuming a rigid wall. A steady laminar flow was assumed because of slow urine flow in the ureter. The pressure at points 1–3 in the renal pelvis relative to the outlet end of the ureter was obtained once the pressure distribution within the renal pelvis and ureter was obtained using CFD (**Figure 1b**), and did not markedly differ among the points (**Figure 1c**). The results showed that the pressure difference within the renal pelvis was uniform.

This study was approved by the Ethics Review Board of Ehime University (no. 2310007). Opt-out informed consent was obtained from the patients for publication of this case report and accompanying images.

## 3 Results

**Figure 2:**
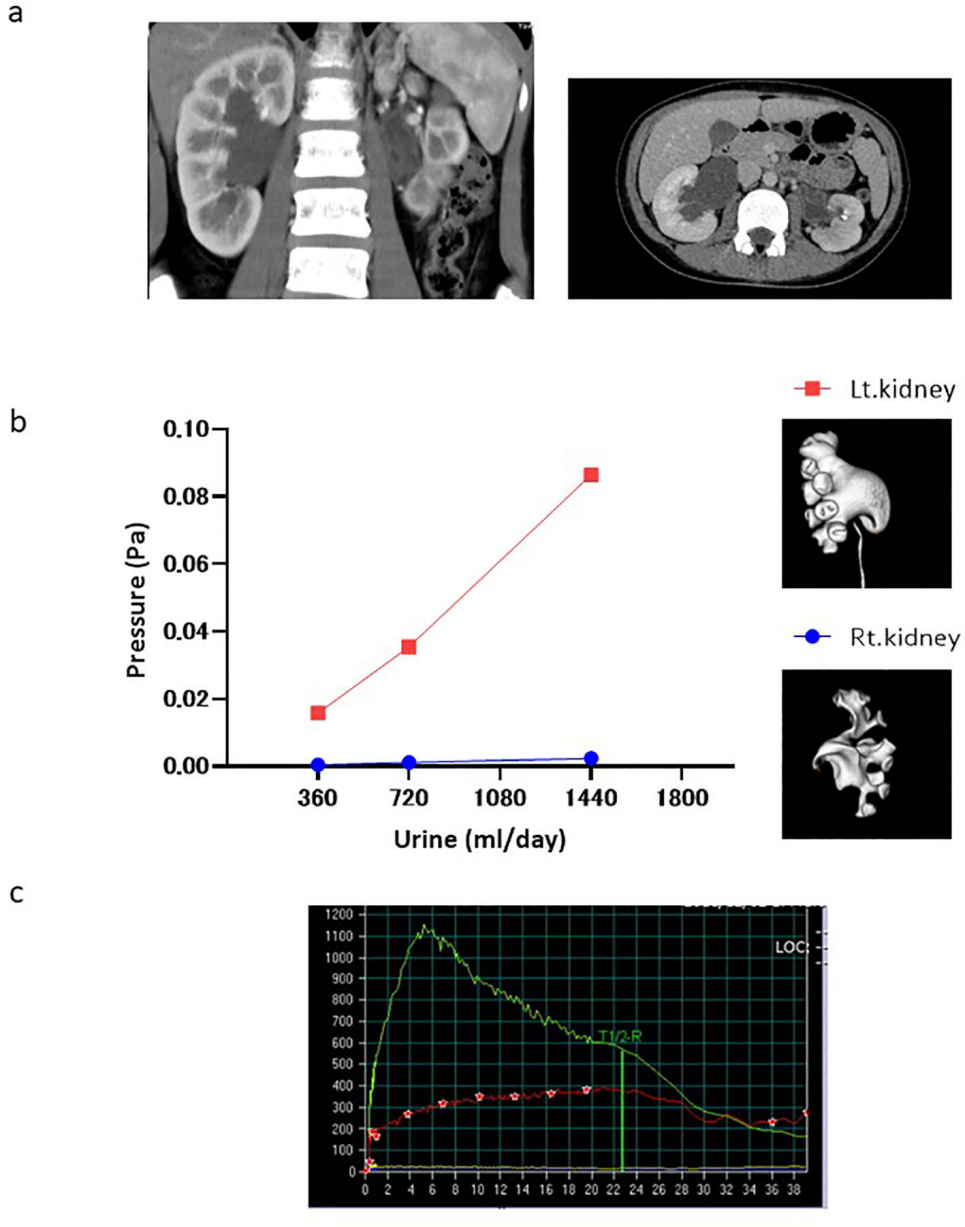
Case presentation. **(a)** Contrast-enhanced CT Tomography. **(b)** Change in pressure difference associated with the virtual urine flow rate in both kidneys (red and blue dots: left and right kidneys, respectively). **(c)** Diuretic renography performed on her for an asymptomatic condition. (Green line: right kidney, red line: left kidney).

This case is an example of elevated renal pelvic pressure revealed by CFD analysis. A 9-year-old girl presented to Ehime University Hospital with left-sided back pain, with a body weight of 35 kg and height of 135 cm. Symptomatic ultrasonography and asymptomatic contrast-enhanced CT revealed bilateral hydronephrosis (left, grades 4 and 1; right, grades 3 and 3) (**Figure 2a**). The virtual urine flow rate setting was changed to 360, 720, and 1440 mL/day, considering her weight, according to the CFD analysis for the left hydronephrosis (grade 1). This increased the pressure difference between the ureter and renal pelvis by 0.015, 0.035, and 0.086 Pa, respectively. However, changes in the pressure difference for right hydronephrosis (grade 3) were not associated with the virtual urine flow rate (**Figure 2b**). A diuretic renogram performed on her for an asymptomatic condition revealed deteriorated left renal function (split renal function, left:right = 24.1%:75.9%) and an obstructive left kidney pattern (**Figure 2c**). Moreover, CT analysis of the left kidney with impaired renal function revealed an elevated renal pelvic pressure. And, the right kidney, which had normal renal function, revealed no elevated pelvic pressure. Thus, hydronephrosis with elevated renal pelvic pressure at physiological urine flow rates suggests that the kidneys were affected.

## 4 Discussion

The two forms of hydronephrosis are differentiated by a renal pelvic pressure that does or does not change with the urine flow rate (11). Pyeloplasty is necessary to prevent renal dysfunction in patients with hydronephrosis and high renal pelvic pressure. However, no imaging method can be used to distinguish the two forms. CFD analysis was used to calculate the pressure difference between the ureter and renal pelvis at a hypothetical urine flow rate. The results of the CT analysis showed that the renal pelvic pressure was elevated in the left kidney with impaired renal function. Thus, hydronephrosis with elevated renal pelvic pressure at physiological urine flow rates may be an indication for pyeloplasty. Moreover, CFD analysis enables more detailed morphological evaluation than ultrasonography by providing a three-dimensional (3D) hydronephrosis model.

**Figure 3:**
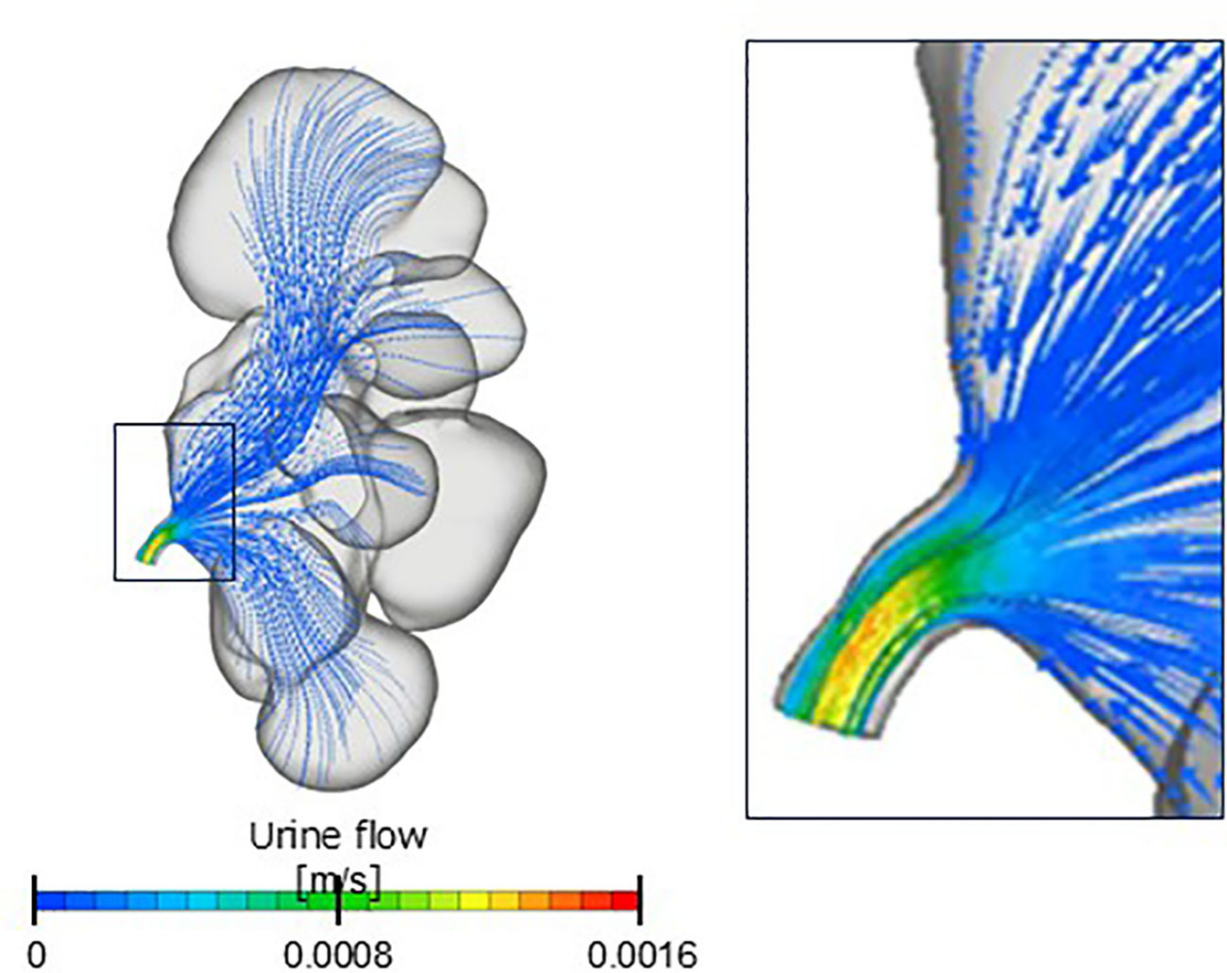
Contour diagram. Renal pelvis flow is a Stokes flow in which vortices and separation do not occur.

We speculate that the pressure difference remains largely consistent regardless of the changes in the hydronephrosis grade, even if CFD analysis is performed on CT scans captured at different time points. This pressure difference likely remains consistent because the flow within the renal pelvis is typically sufficiently slow to be approximated as Stokes flow, where vortices and separation do not occur. The renal pelvic pressure was almost uniform, indicating minimal or no pressure loss in that region (**Figure 3**). Therefore, we believe that the pressure difference remained unchanged, as determined by the morphology of the ureteropelvic junction, rather than that of the renal pelvis.

This study has some limitations. First, the intrarenal pressure analyzed using CFD is an estimate and not an actual pressure. Therefore, the pressure estimated using the CFD must be verified using the Whitaker test. Second, constructing a 3D hydronephrosis model that can be analyzed using plain CT is challenging. Thin-slice imaging data from contrast-enhanced computed tomography (CT) are required to create a 3D hydronephrosis model for CFD analysis. Finally, this was a retrospective study; further prospective studies are required to validate our findings. A long-term follow-up prospective study incorporating CFD analysis should be conducted to evaluate trends in renal function in patients with hydronephrosis.

## 5 Conclusions

Our preliminary findings suggest that CFD analysis can be used for estimating the increase in renal pelvic pressure and predicting renal function deterioration. In the future, CFD analysis can be used to evaluate severity of obstruction.

## Data Availability

The raw data supporting the conclusions of this article will be made available by the authors, without undue reservation.
